# Perceptions of childhood immunization in São Paulo: quantitative-qualitative cross-sectional study

**DOI:** 10.1590/1516-3180.2023.0447.R1.05062024

**Published:** 2024-10-21

**Authors:** Lucas de Brito Costa, Carolina Nunes França, Luiz Henrique da Silva Nali, Patrícia Colombo-Souza, Neil Ferreira Novo, Yára Juliano

**Affiliations:** I Master’s student, Postgraduate Program in Health Sciences, Universidade de Santo Amaro (UNISA), São Paulo, Brazil.; II Postgraduate Program Coordinator, Postgraduate Program in Health Sciences, Universidade de Santo Amaro (UNISA), São Paulo, Brazil.; III Professor, Postgraduate Program in Health Sciences Universidade de Santo Amaro (UNISA), São Paulo, Brazil.; IV Professor, Postgraduate Program in Health Sciences Universidade de Santo Amaro (UNISA), São Paulo, Brazil.; V Professor, Postgraduate Program in Health Sciences, Universidade de Santo Amaro (UNISA), São Paulo, Brazil.; VI Professor, Post-Graduation Program in Health Sciences, Santo Amaro University, São Paulo, Brazil.

**Keywords:** Vaccination., Immunization., Vaccine refusal., Child., COVID-19., Vaccine-preventable diseases., Vaccination coverage., Anti-vaccine movements.

## Abstract

**BACKGROUND:**

Vaccination hesitation spans from historical diseases such as smallpox to the current challenges with the coronavirus disease (COVID-19). In Brazil, vaccination faces obstacles related to trust and convenience. Despite the National Immunization Program, fear of adverse effects as well as misinformation challenge confidence in vaccines, and anti-vaccine movements have gained momentum.

**OBJECTIVES:**

This study investigated childhood vaccine refusal, including COVID-19 vaccines, by comparing the reasons for and sociodemographic differences between vaccinated individuals and those who hesitated or refused immunization.

**DESIGN AND SETTING:**

A cross-sectional study was conducted in São Paulo, Brazil, using questionnaires administered during pediatric consultations between January and April 2023.

**METHODS:**

This study investigated vaccine hesitancy and the attitudes of parents and caregivers of children (0–12 years) towards vaccines. The questionnaire was administered during routine pediatric consultations at three different locations, each with 50 participants for a total of 150 participants, to avoid selection bias.

**RESULTS:**

Marked differences were evident among caregivers in terms of sex, race, income, education, and religion, which influenced their attitudes toward vaccination. There was an increase in the refusal of seasonal vaccinations and a significant distrust of the efficacy of the COVID-19 vaccine (52%), with concerns about its side effects. Although most patients did not stop vaccination, significant delays occurred, especially in the clinical setting (58%).

**CONCLUSIONS:**

This study emphasizes the importance of childhood health decisions, indicating the need to build trust in vaccines, tailor health policies, and investigate the causes of distrust to promote childhood immunizations.

## INTRODUCTION

Issues of vaccine hesitancy are not exclusive to the 21st century. They have unfolded throughout history, from the smallpox vaccine to the measles, mumps, and rubella (MMR) vaccine in the 20th century, the human papillomavirus vaccine, and currently, the coronavirus disease (COVID-19) vaccine. Vaccination is the most effective way to prevent infectious diseases and has been responsible for completely eradicating smallpox and significantly reducing cases of measles, polio, and tetanus in many parts of the world.^1,2^ Despite overwhelming evidence of vaccine effectiveness and safety, an increasing number of people hesitate to receive recommended vaccines or refuse them altogether.^2^ It is a serious issue because it is linked to the resurgence of vaccine-preventable diseases, such as measles, in the United States and Europe. In Brazil, vaccination coverage for MMR has consistently decreased since 2013, raising concerns about increasing rates of unvaccinated individuals nationwide. This increases the risk of new disease outbreaks, which can be prevented using vaccines. In this context, understanding the vaccine confidence in Brazil is more crucial than ever.^3^

Figueiredo *et al.* ^4^ conducted a qualitative study involving interviews with families having children under 2 years old. Throughout these interviews, several barriers and influencing factors regarding vaccination were identified, addressing issues related to convenience, trust, and concerns about administering multiple vaccines simultaneously. Barbieri *et al.* ^5^ also conducted a study with middle-class parents in São Paulo and concluded that parents vaccinating their children felt part of Brazil’s “immunization culture,” while those refusing vaccination felt that mandatory vaccination was incompatible with their lifestyle.

The Brazilian National Immunization Program (PNI) of the Pan American Health Organization (PAHO), a branch of the World Health Organization (WHO), has been cited as a global reference. Established by Law 6.259/75, this program provides 50 immunobiological products (serums, vaccines, and immunoglobulins) free of charge to all inhabitants of the Brazilian territory, including 33 vaccines, 19 of which are part of the National Vaccination Schedule to prevent over 20 infectious diseases across various age groups. Additionally, 10 special vaccines are available at Special Immunobiological Reference Centers (CRIEs) for groups with specific clinical conditions, such as people living with HIV.^6,7^

In this scenario, the fear of adverse events and the circulation of false information about immunobiological products overshadow knowledge about the importance and benefits of vaccines. Although not highly active in Brazil, antivaccine movements are becoming more frequent and persuasive, disseminating scientifically unfounded information on vaccine risks.

## OBJECTIVE

This study aimed to analyze the frequency of vaccine refusal among parents and guardians regarding the available immunizations for the pediatric population, including those intended for COVID-19, to be administered to their children and/or wards in São Paulo, Brazil.

## METHODS

This was a quantitative-qualitative cross-sectional study conducted in the city of São Paulo between January and April 2023, focusing on parents and caregivers of patients aged 0–12 years who attended routine pediatric consultations. Using the validated “Parental Attitudes on Childhood Vaccines” questionnaire, the study investigated vaccine hesitancy and caregivers’ willingness to vaccinate their children, addressing vaccination behavior, safety, and confidence in vaccines. Sociodemographic information was collected and the research was conducted in three distinct locations within the same region of São Paulo city (Universidade Santo Amaro, UNISA; Basic Health Unit, BHU; and Medical Office), each with 50 participants, totaling 150, to mitigate selection bias.

### Ethical Considerations

The study design and methodology were approved by the Research Ethics Committee of Santo Amaro University (5,770,898) on November 22, 2022. The study was conducted in accordance with the principles of the Declaration of Helsinki, and informed consent was obtained from all participants before the study commenced.

### Statistical analysis

Categorical variables were analyzed using the chi-squared test. Statistical significance was set at P < 0.05.

## RESULTS

In the study, a significant predominance of the female sex (82.7%) among the caregivers was observed across all settings, compared to male caregivers (chi-square test X2 = 10.14, P = 0.0063). Concerning race/ethnicity, the BHU and UNISA settings exhibited similarities, with a prevalence of Black and mixed-race individuals, whereas the Medical Office had a majority of White individuals (46%), which was a statistically significant difference (P < 0.0001, X2 = 40.43). Regarding family income, a higher percentage of families fell within the ranges of up to one minimum wage and two to four minimum wages compared to the group earning five to ten minimum wages, with statistically significant differences (P < 0.0001, X2 = 75.40). In terms of education, the Medical Office showed more caregivers with incomplete college education (36%), while BHU and UNISA had the majority with completed high school education (36% and 38%, respectively), with statistically significant differences (P < 0.0001, X2 = 115.0). Concerning religion, there was a predominance of Christian denominations across all settings, with Protestants (82%), followed by Catholics (60%), showing no statistically significant difference (P = 0.3910, X2 = 12.70). Additionally, the relationship between parents/guardians and partners was also significant (P = 0.0147, X2 = 9.44) (**Table 1**).

**Table 1:** Characterization of research participants according to demographic variables and study setting

	BHU		UNISA		Medical office		Total	
	n	%	n	%	n	%	n	%
**Sex**								
Female	47	94	42	84	35	70	124	82,7
Male	3	6	8	16	15	30	26	17,3
Total	50	100	50	100	50	100	150	100
**Race**								
White	8	16	9	18	23	46	40	26,7
Black	19	38	21	42	7	14	47	31,3
Mixed race	20	40	15	30	4	8	39	26
Yellow/indigenous	3	6	5	10	16	32	24	16
Total	50	100	50	100	50	100	150	100
**Family income**								
Up to 1 minimum wage	33	66	32	64	0	0	65	43,3
2 to 4 minimum wages	17	34	15	30	25	50	57	38
5 to 10 minimum wages	0	0	3	6	25	50	28	18,7
Total	50	100	50	100	50	100	150	100
**Education**								
Incomplete elementary school	6	12	1	2	0	0	7	4,6
Complete elementary school	16	32	11	22	0	0	27	18
Incomplete high school	9	18	18	36	0	0	27	18
Complete high school	18	36	19	38	11	22	48	32
Incomplete college	1	2	1	2	18	36	20	13,4
Complete college	0	0	0	0	14	28	14	9,4
Incomplete postgraduate	0	0	0	0	7	14	7	4,6
Total	50	100	50	100	50	100	150	100
**Religio**n								
Catholic	18		21		21		60	
Protestant	31		27		24		82	
Buddhist	0		1		0		1	
Jewish	0		0		1		1	
Umbanda	1		0		0		1	
Spiritist	0		0		1		1	
Atheist	0		1		3		4	
Total	50		50		50		150	
**Marital status**								
With partner	24	48	29	58	38	76	91	60,7
Without partner	26	52	21	42	12	24	59	39,3
Total	50	100	50	100	50	100	150	100

BHU = basic health unit; UNISA = Universidade Santo Amaro.

The results presented in **Table 2** show an increasing refusal of seasonal vaccinations for the flu, measles, and yellow fever. Refusal of the human rotavirus vaccine is influenced by age restrictions. Some children did not receive more than one vaccine, thereby increasing the absolute refusal rates. Although not statistically significant, there was a trend towards higher refusal rates in clinical settings than in the other studied scenarios (X2 = 9.22, P = 0.3240).

**Table 2:** Acceptance or refusal of vaccination by available immunization in the National Immunization Program

	BHU		UNISA		Medical Office		TOTAL	
	n	%	n	%	n	%	n	%
**VACCINES**								
Bcg	0	0	0	0	0	0	0	0
Hepatitis	0	0	0	0	0	0	0	0
Pneumo 10	0	0	0	0	0	0	0	0
Pentavalent	0	0	0	0	0	0	0	0
Polio	0	0	0	0	0	0	0	0
Rotavírus	0	0	1	25	1	2,1	2	2,9
Meningococcal c	0	0	0	0	0	0	0	0
Measles, mumps, rubella (MMR)	0	0	0	0	0	0	0	0
Influenza	8	42,1	2	50	18	36,7	28	41,7
Yellow fever	1	5,2	0	0	4	8,2	5	7,4
Measles	0	0	0	0	0	0	0	0
Sarampo	2	10,6	0	0	4	8,2	6	8,9
Human papillomavirus (HPV)	0	0	0	0	0	0	0	0
COVID-19	8	42,1	1	25	22	44,8	31	46,2
**Total**	19	100	4	100	49	100	67	100

BHU = basic health unit; UNISA = Universidade Santo Amaro; COVID-19 = coronavirus disease.

Regarding the reasons for refusing childhood vaccination, as shown in **Table 3**, the majority of respondents at BHU (60%) and UNISA (82%) denied having any concerns. However, in the medical office, the primary reason for refusal was distrust of the vaccine efficacy, which reached 52%. This data was significant, with X2 = 47.33 and P < 0.0001. Regarding immunization delays, 58% of caregivers at the medical office admitted to delays, while the overall average was 31%. The most significant delays occurred in the medical office (58%), followed by BHU and UNISA (26% and 10%, respectively), with X2 = 27.76 and P < 0.0001 in the chi-square test for this combined analysis.

**Table 3:** Reliability and vaccine refusal by available immunization in the National Immunization Program

	BHU		UNISA		Medical Office		TOTAL	
	n	%	n	%	n	%	n	%
**Trust**								
No fear	30	60	41	82	12	24	83	55,3
Fearful	12	24	9	18	12	24	33	22
Does not believe	8	16	0	0	26	52	34	22,7
Total	50	100	50	100	50	100	150	100
**Vaccination delay**								
Yes	13	26	5	10	29	58	47	31,3
No	37	74	45	90	21	42	103	68,7
Total	50	100	50	100	50	100	150	100

BHU = basic health unit; UNISA = Universidade Santo Amaro.

In this study, amid the pandemic context, specific apprehensions regarding the coronavirus vaccine were also assessed, as shown in **Table 4**. At the private healthcare office, 74% of guardians of children aged 0–12 years expressed some level of concern about this particular vaccine, which can be explained by fear of side effects and disbelief in vaccine efficacy. The chi-square test for this analysis resulted in X2 = 32.31 and P < 0.0001. Concerning the COVID-19 vaccine, a high rate of complete administration of the available doses was observed at the BHU and an outpatient clinic linked to an educational institution. However, more than half of the patients in private medical offices (56%) completed the vaccination schedule. The chi-square test for this aspect resulted in X2 = 25.19 and P < 0.0001.

**Table 4:** Apprehension towards coronavirus disease vaccine and vaccination of children with available doses

	BHU		UNISA			Medical office		TOTAL	
	n	%	n	%	n	%	n	%
**Apprehension for COVID-19 shots**								
Yes	20	40	9	18	37	74	66	44
No	30	60	41	82	13	26	84	56
Total	50	100	50	100	50	100	150	100
**Has recieved all COVID-19 doses**								
Yes	42	84	49	98	28	56	119	79,3
No	8	16	1	2	22	44	31	20,7
Total	50	100	50	100	50	100	150	100

BHU = basic health unit; UNISA = Universidade Santo Amaro; COVID-19 = coronavirus disease.

At the end of the questionnaire, parents and/or caregivers were asked whether they had missed vaccinating children under their care at any point. The results, shown in **Table 5**, indicate that a significant majority of those responsible for children at the BHU (84%) and UNISA (96%) stated that they had not missed vaccinating their children. In these results, it is important to note a distinction regarding vaccination delays. While delays still allowed for the possibility of updating overdue vaccines, this result considered only vaccines that were not offered or for which there was no longer an interest in receiving them. The statistical analysis for this question resulted in X2 = 27.89 and P < 0.0001.

**Table 5:** Overall outcome of vaccine refusal

	BHU		UNISA		Medical Office		TOTAL	
	n	%	n	%	n	%	n	%
**Have missed/delayed any vaccine shot?**								
Yes	8	16	2	4	22	44	31	20,7
No	42	84	48	96	28	56	118	78,7
Total	50	100	50	100	50	100	150	100

BHU = basic health unit; UNISA = Universidade Santo Amaro.

## DISCUSSION

The present study highlights the predominance of female caregivers across all settings, underscoring socioeconomic and educational differences. There has been a growing refusal of seasonal vaccinations, the influence of age restrictions on refusal of the human rotavirus vaccine, and a tendency toward higher refusal rates in clinical settings. The diverse reasons for refusal included distrust of the vaccine efficacy, notably in the medical office. Immunization delays were more frequent in medical offices, and reasons for hesitation regarding the coronavirus vaccine were emphasized. Various rates of complete administration of the COVID-19 vaccine were observed, while the majority of caregivers in healthcare units reported not having missed vaccinating their children, considering that vaccines were not offered, or lacking interest.

The predominance of women as caregivers across various environments suggests significant cultural and social influences, highlighting the need for a more detailed investigation of their impact on decisions concerning children’s health. This finding aligns with a study conducted in Fortaleza, Ceará, which identified a predominance of females in caregiving roles, mainly mothers as the primary or sole caregivers of their children.^8^ The racial disparity observed in different settings, with a higher representation of Black and mixed-race individuals in BHU and UNISA, and the prevalence of White individuals in private healthcare, underscore the influence of socioeconomic conditions on accessibility to healthcare services. The correlation between caregivers’ family income and education in specific settings suggests the need for more comprehensive healthcare policies that cater to diverse socioeconomic and educational realities. Although the prevalence of Christianity across all settings did not demonstrate a statistically significant difference among religious groups, cultural aspects and beliefs may influence attitudes toward health and vaccination. It is crucial to explore how these factors impact adherence to immunization practices and preventive care; in some cases, non-vaccination is more closely related to healthcare service characteristics than to specific populations.^4^

The analysis revealed a progressive increase in refusal of seasonal vaccinations against influenza, measles, and yellow fever. This was particularly evident in the refusal of the human rotavirus vaccine, which was largely influenced by age-related restrictions. Furthermore, in some cases, children did not receive more than one vaccine, contributing to an absolute increase in refusal rates. In the study by Figueiredo et al.^4^, one of the reasons for refusal was a lack of knowledge about the currently available vaccines. Interestingly, although not statistically significant, there was a tendency for higher refusal rates in clinical settings than in other analyzed contexts. This trend underscores the need to better understand the factors that influence vaccine acceptance or refusal, particularly in clinical settings, and to implement strategies that promote greater adherence to vaccination.

When analyzing the reasons for childhood vaccination refusal, different perspectives emerged across the healthcare settings under investigation. In the BHUs and at UNISA, the majority of respondents denied refusal, with rates of 60% and 82%, respectively. However, in medical offices, distrust of vaccine efficacy was identified as the primary cause, affecting 52% of the refusal decisions. The issue of mistrust aligns with the findings of another study conducted in São Paulo, which highlighted that major reasons for hesitancy were linked to trust, convenience, complacency, and other unknown reasons. The majority of doubts stemmed from trust issues.^3^ In a 2017 technical report from the European Centre for Disease Prevention and Control, results showed vaccine hesitancy due to lack of trust, complacency, and vaccine convenience, labeled as the 3C model.^9^

Additionally, when exploring the issue of immunization delays, significant disparities were observed among different settings. In the medical office, 58% of caregivers admitted to delays, contrasting with the lower averages of 26% and 10% in the UBS and UNISA, respectively. This discrepancy underscores the need for targeted strategies to mitigate immunization delays, especially in clinical contexts where the prevalence of these delays has been shown to be more prominent.^5^ In countries such as Italy, where childhood vaccination rates are decreasing, the strategy adopted involves stricter laws affecting school admission.^10^

Vaccine hesitancy is not a new phenomenon, yet the specific concern regarding the coronavirus vaccine amid the pandemic might have escalated as vaccine acceptance is higher when there is confidence in its effectiveness and safety,^9^ something not witnessed in Brazil. Vaccine hesitancy during the COVID-19 pandemic may not be directly comparable to that in previous contexts. No virus in recent memory has so broadly disrupted social life and society as COVID-19.^11^ However, in the clinic, a significant 74% of caregivers expressed concern about this vaccine, possibly linked to fear of side effects and distrust in vaccine effectiveness, cited as primary reasons for vaccination refusal or hesitation within this group. There was a disparity in the complete administration rate of COVID-19 vaccine doses among healthcare settings. While UBS and UNISA showed high completion rates, only about 56% of the patients at the clinic completed the process. In particular, compliance with containment measures might depend on the degree of trust in authorities and other healthcare services,^3,12^ and fear of consequences, such as reporting to child protective services.^5^

Upon completing the data collection through questionnaires, parents and/or caregivers were asked about the potential cessation of vaccination in children under their care. The results revealed that a significant majority of guardians in the settings of the BHU and UNISA (84% and 96%, respectively) stated that they had not ceased vaccinating their children. It is relevant to note that this analysis distinguished itself from vaccination delays, focusing solely on situations where vaccines were not offered or when there was no interest in receiving them. This differentiation is crucial for understanding the dynamics between the explicit refusal of certain vaccines and delays, providing valuable insights for directing more effective strategies in the context of childhood vaccination.

It is of utmost importance to emphasize that socioeconomic status is a major issue that may impact hesitancy. Previous studies have described important inequalities that may imply access, decision, or delay in receiving the vaccination^13,14^. Since the Brazilian National Vaccination Program provides free vaccination to children on its schedule, lack of access does not seem to be a major problem but may be associated with misinformation that might be stimulated by the influence of social media^15^, which may directly impact the perceived reliability of the vaccines. Regardless of the findings described on vaccines, fear of side effects, distrust of political involvement, and underestimation of the severity of infectious diseases, such as the COVID-19 pandemic are still frequent and present within the population^16,17^. Therefore, the implementation of strategic public health policies combined with well-planned and comprehensive vaccination campaigns is mandatory to reduce vaccination hesitancy and achieve greater vaccination coverage against COVID-19, and this should be applied to all other vaccines.

## CONCLUSION

This study underscores the relevance of childhood healthcare decisions and their socioeconomic impact. This revealed vaccine hesitancy, particularly in clinical settings, emphasizing the importance of approaches to foster greater trust and information. These findings highlight the need for more inclusive healthcare policies tailored to diverse socioeconomic realities. An in-depth investigation into the roots of this hesitancy, considering cultural and demographic nuances, is necessary to achieve a comprehensive understanding of attitudes toward childhood vaccination. Furthermore, exploring specific strategies to enhance vaccine acceptance and adherence in various clinical contexts may be pivotal for advancing public health and childhood immunization.
